# Comprehensive analysis on subchondral bone marrow lesions of human osteoarthritis by integrating bulk and single-cell transcriptomes

**DOI:** 10.1186/s12891-023-06676-4

**Published:** 2023-08-25

**Authors:** Muhui Zeng, Xiaoshuai Wang, Tianyu Chen, Guangfeng Ruan, Jia Li, Song Xue, Yang Zhao, Zhiyang Hu, Ye Xie, Tianxiang Fan, Shibo Chen, Yang Li, Qianyi Wang, Yue Zhang, Rongkai Zhang, Lijun Lin, Changhai Ding, Zhaohua Zhu

**Affiliations:** 1 Clinical Research Centre, Zhujiang Hospital, Southern Medical University, Guangzhou, 510280 Guangdong China; 2 Department of Orthopedics, General Hospital of Southern Theater Command of PLA, Guangzhou, Guangdong China; 3 Department of Orthopedics, Zhujiang Hospital, Southern Medical University, Guangzhou, Guangdong China; 4 Department of Orthopedics, The Third Affiliated Hospital of Southern Medical University, Guangzhou, China; 5 Clinical Research Centre, Guangzhou First People’s Hospital, School of Medicine, South China University of Technology, Guangzhou, China; 6 Department of Rheumatology and Immunology, Arthritis Research Institute, The First Affiliated Hospital of Anhui Medical University, Hefei, China; 7 Sun Yat-sen University School of Medicine, Sun Yat-sen University, Shenzhen, China; 8 Zhongshan School of Medicine, Sun Yat-sen University, Guangzhou, China; 9 State Key Laboratory of Organ Failure Research, Department of Cell Biology, School of Basic Medical Sciences, Southern Medical University, Guangzhou, China; 10 Menzies Institute for Medical Research, University of Tasmania, Hobart, Tasmania Australia

**Keywords:** Osteoarthritis, Subchondral bone, Bone marrow lesions, Deconvolution algorithm, Single-cell RNA sequencing

## Abstract

**Objective:**

This study aims to demonstrate the cellular composition and underlying mechanisms in subchondral bone marrow lesions (BMLs) of knee osteoarthritis (OA).

**Methods:**

BMLs were assessed by MRI Osteoarthritis Knee Score (MOAKS)≥2. Bulk RNA-sequencing (bulk-seq) and BML-specific differentially expressed genes (DEGs) analysis were performed among subchondral bone samples (including OA-BML=3, paired OA-NBML=3; non-OA=3). The hub genes of BMLs were identified by verifying in independent datasets and multiple bioinformatic analyses. To further estimate cell-type composition of subchondral bone, we utilized two newly developed deconvolution algorithms (MuSiC, MCP-counter) in transcriptomic datasets, based on signatures from open-accessed single-cell RNA sequencing (scRNA-seq). Finally, competing endogenous RNA (ceRNA) and transcription factor (TF) networks were constructed through multiple predictive databases, and validated by public non-coding RNA profiles.

**Results:**

A total of 86 BML-specific DEGs (up 79, down 7) were identified. *IL11* and *VCAN* were identified as core hub genes. The “*has-miR-424-5p/lncRNA PVT1”* was determined as crucial network, targeting *IL11* and *VCAN*, respectively. More importantly, two deconvolution algorithms produced approximate estimations of cell-type composition, and the cluster of heterotopic-chondrocyte was discovered abundant in BMLs, and positively correlated with the expression of hub genes.

**Conclusion:**

*IL11* and *VCAN* were identified as the core hub genes of BMLs, and their molecular networks were determined as well. We profiled the characteristics of subchondral bone at single-cell level and determined that the heterotopic-chondrocyte was abundant in BMLs and was closely linked to *IL11* and *VCAN*. Our study may provide new insights into the microenvironment and pathological molecular mechanism of BMLs, and could lead to novel therapeutic strategies.

**Supplementary Information:**

The online version contains supplementary material available at 10.1186/s12891-023-06676-4.

## Key points

1. *IL11* and *VCAN* were identified as the core hub genes of BMLs in OA.

2. The “*has-miR-424-5p/lncRNA PVT1”* and the “*3,4-benzopyrene”* were determined as crucial networks and ingredient targeting *IL11* and *VCAN*, respectively.

3. The cluster of heterotopic-chondrocytes was determined abundant in BMLs by deconvoluting from scRNA-seq to bulk-seq, and positively correlated with the expression of hub genes.

**Supplementary Information:**

The online version contains supplementary material available at 10.1186/s12891-023-06676-4.

## Introduction

Osteoarthritis (OA) is a prevalent aging and degenerative disease that causes restricted function and persistent pain, and reduces the life quality for millions of individuals worldwide [1]. OA is considered as a whole joint illness involving several structural abnormalities including cartilage loss, subchondral bone marrow lesions (BMLs), ligament tears, synovitis, and periarticular muscle weakness [2]. In recent years, there is a growing body of evidence that BMLs might be the early lesions of joint damage in OA with emerging research on their histology and histomorphometry [3–5]. Patients with worsening BMLs have been identified as being at high risk for OA progression [6]. The BMLs are characterized by regions of bone resorption or sclerosis, inflammation, angiogenesis and de-novo cartilage [7] histologically, and visualized as hypointense signal alterations on T1-weighted Magnetic Resonance Imaging (MRI) and hyperintense signal alterations on T2-weighted fat-suppressed MRI [8].

Though several researches have reported the pathologic and genetic changes of subchondral BMLs in OA [9–13], there are still large uncharted territories. First, although the differentially expressed genes (DEGs) between OA-BML tissue and non-OA tissue were identified, the differences in gene expression between OA non-BMLs (OA-NBML) tissue and OA-BML tissue have not yet to be described. Second, the pathological changes in OA-BML, such as imbalance of osteoblasts, osteoclasts, were described [14]. However, the cellular microenvironment of subchondral bone was so far absent. Importantly, the single-cell RNA sequencing (scRNA-seq) could be useful for uncovering the complexities of the molecular components of BMLs. Interestingly, there are series of algorithms that use deconvolution approaches to characterize cell-type compositions at the single-cell level, such as MUlti-Subject SIngle Cell deconvolution (MuSiC) [15] and Microenvironment cell population (MCP)-counter [16]. These algorithms are able to map the cell type-specific gene expression information from scRNA-seq to bulk RNA-sequencing (bulk-seq) [17]. Last, the association between crucial genes of subchondral BMLs and OA biological processes are still lacking, without identifying hub genes (the most important genes among the DEGs) as well. Moreover, the epigenetic regulatory mechanism of BMLs, for examples, “mRNA-TF (transcript factor)” network [18] and competing endogenous RNA (ceRNA) network [19], are also unknown.

In this study, we aimed to integrate the analysis of bulk-seq and scRNA-seq data to unravel both the cellular atlas and underlying mechanisms of subchondral BMLs in knee OA.

## Materials and methods

### Study design and human subchondral bone samples collection

**Fig. 1:**
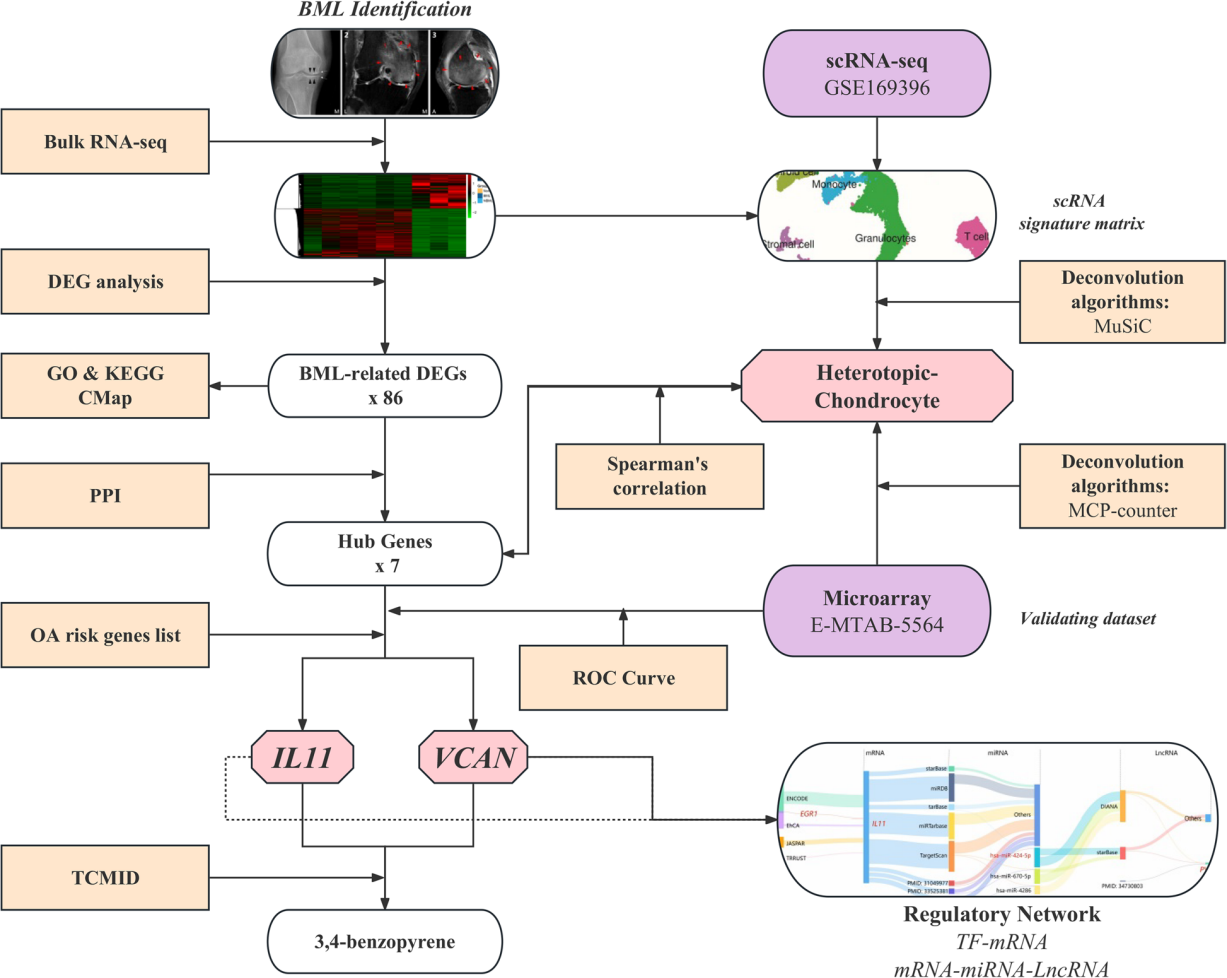
Schematic diagram of study design. Subchondral bone samples were collected from OA patients with or without MRI detected BML, as well as from non-OA post-mortem cases. RNA-seq was applied to map the gene expression patent among subchondral bone BML. IL11 and VCAN were identified and validated as core hub genes of BML by using DEG and PPI analysis. Besides, BML-abundant cell types were defined by integrating bulk and public scRNA-seq data (GSE169396) and validated by external dataset (E-MTAB-5564). Subsequently, the correlations between cell composition and hub genes were also analyzed. Additionally, the regulatory networks of IL11 and VCAN were constructed. Finally, 3,4 benzopyrene was predicted as the potential therapeutic drug for BMLs by targeting IL11 and VCAN. Abbreviation: BML, Bone Marrow Lesion; NBML, Non Bone Marrow Lesion; DEG, Differential Expression Gene; GO, Gene Ontology; KEGG, Kyoto Encyclopedia of Genes and Genomes; PPI, Protein–Protein Interaction; CMap, Connectivity Map; TCMID, Traditional Chinese Medicine Integrated Database; ROC, Receiver Operating Characteristic; scRNA-seq, single-cell RNA sequencing; MuSiC, MUlti-Subject SIngle Cell deconvolution; MCP-counter, Microenvironment Cell Population counter; TF, Transcript factor

**Fig. 2:**
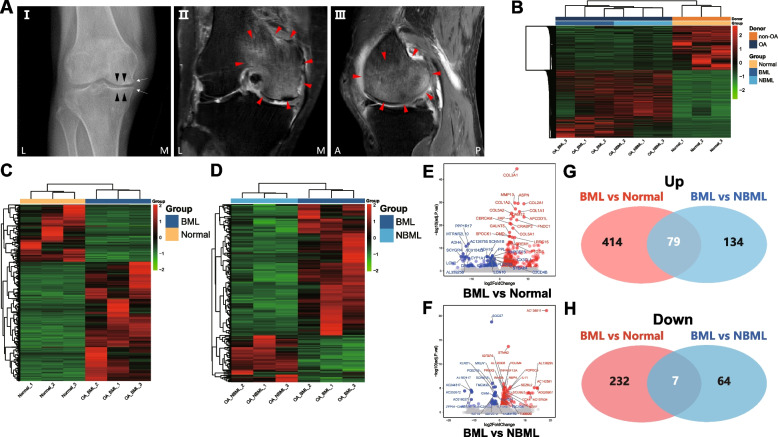
Bulk RNA-sequencing analysis of subchondral bone tissues. **A** Representative characteristics of subchondral bone sample from OA donor (Female, 80y) receiving total knee replacement surgery (I) Definitive diagnosis of OA by knee radiograph (Posteroanterior, K-L Grade=2). White arrow, osteophyte; black arrowhead, joint space narrowing. T2-weighted MRIs, coronal (II) and sagittal (III) plane with BML (Red arrowhead), were evaluated by MOAKS (Grade 3). **B**-**D** Unsupervised hierarchical clustering was performed and visualized by heatmap. Red color represents upregulated genes and green color represents the opposite. Distances between samples were detected using the “Euclidean” algorithm and clustered with the “single linkage” method. **B** The OA samples (orange) were clustered together and separated from the non-OA samples (deep blue). The OA-BML samples (deep blue) were clustered together and distinguished with **C** healthy samples (yellow) or **D** OA-NBML samples (light blue). **E**-**F** Respective volcano plot of the two comparisons: **E** OA-BML vs non-OA; **f** OA-BML vs OA-NBML. Red dots represented genes with Fold change (FC) > 1.5 (blue dots represented FC<1.5 , and adjusted *p* value < 0.05, while. Grey dots represented the remaining genes without significant difference. **G**-**H** Venn diagrams visualized individual and common upregulated (**G**) and downregulated (**H**) DEGs in (OA-BML vs non-OA) and (OA-BML vs OA-NBML)

The schematic diagram of study design is shown in Fig. 1. Samples from six participants without history of knee injury, surgery, rheumatoid arthritis or pseudogout were obtained, including three female OA patients (mean ± standard deviation [SD] age, 72.0 ± 8.5 years) who underwent total knee replacement (TKR) surgery, and three non-OA post-mortem cases (mean ± standard deviation [SD] age, 30.3 ± 8.3 years). Informed consents were obtained from all donors, and ethical approval was granted by the medical ethics committee of Zhujiang Hospital of Southern Medical University (2019-KY-073-01). Radiographic OA was confirmed by knee joint X-ray images (Kellgren-Lawrence grade≥2), and the inclusion and exclusion criteria for OA patients were based on American College of Rheumatology classification criteria for OA. Each region of subchondral BMLs was identified by 3.0T MRI (Philips) (Fig. 2A). T2-weighted sequences were obtained to evaluate the BMLs by MRI Osteoarthritis Knee Score (MOAKS) system [8]. The grade of each region (volume: grade 0 = none, grade 1 <33%, grade 2 = 33~ 66%, and grade 3 >66%) was accessed by experienced radiologists. The presence of BML was determined as grade ≥ 2. The adjacent regions without BMLs were collected as paired non-BML (NBML) samples, which were also identified by MRI.

### Subchondral bone cells collection

Human subchondral bone samples were collected from TKR surgeries and post mortem cases. Bone samples were cut into 1–2 mm pieces and then digested in 1% type IA collagenase (C9891-1G, Sigma) for 3 hours [20]. Cells were then collected after red blood cell lysis.

### Bulk RNA-sequencing

#### RNA extraction

Total RNA was isolated using trizol reagent (Invitrogen, Carlsbad, CA, USA). Next, the RNA quality and purity were checked by NanoDrop ND-1000 (NanoDrop, Wilmington, DE, USA). The RNA integrity was assessed by Bioanalyzer 2100 (Agilent, CA, USA) with RIN number >7.0, and confirmed by electrophoresis with denaturing agarose gel. Through two rounds of purification, Poly (A) RNA was purified from 1μg total RNA using Dynabeads Oligo (dT)25-61005 (Thermo Fisher, CA, USA). Then the poly (A) RNA was fragmented into small pieces using Magnesium RNA Fragmentation Module (NEB, cat.e6150, USA) under 94℃ (5-7min).

#### Library construction

First, the cleaved RNA fragments were reverse-transcribed to create the cDNA by SuperScript™ II Reverse Transcriptase (Invitrogen, cat. 1896649, USA). Second, it was used to synthesize U-labeled second-stranded DNAs with E. coli DNA polymerase I (NEB, cat.m0209, USA), RNase H (NEB, cat.m0297, USA), and dUTP Solution (Thermo Fisher, cat.R0133, USA). Third, an A-base was added to the blunt ends of each strand, preparing them for ligation to the indexed adapters. After the heat-labile UDG enzyme (NEB, cat.m0280, USA) treatment of the U-labeled second-stranded DNAs, the ligated products were amplified with PCR as conventional conditions. Finally, the average insert size for the final cDNA library was 300±50 bp.

#### Sequencing

We performed the 2×150 bp paired-end sequencing (PE150) on an llumine Novaseq™ 6000 (LC-Bio Technology CO., Ltd., Hangzhou, China) following the vendor’s recommended protocol [21]. The Dataset and detailed information are available in NCBI under the accession number PRJNA800573.

### Identification of BML-specific differentially expressed genes (DEGs)

#### Analysis of differential gene expression and hierarchical clustering

Differential expression analysis was performed between subchondral bone samples using DESeq2 R package(ver.1.30.1). The Benjamini-Hochberg method was used to correct for multiple testing with a significance cutoff value of 0.05 as indicated by the adjusted *P*-value [22]. In short, we selected the differentially expressed genes based on the following criteria: |Fold change (FC)|>1.5, |log_2_FC| > 0.585, and adjusted *P* value < 0.05.

DEGs were selected for subsequent clustering. Hierarchical clustering was performed by “hclust” function in R. Heatmaps were used to visualize the expression level of shared DEGs between OA and non-OA groups, OA-BML and OA-NBML groups, and were plotted according to hierarchical clustering analysis (Supplementary Table S2). Volcano plots and heatmaps were generated using “ggplot2” (ver. 3.3.2) and “pheatmap” (ver. 1.0.12) in R.

### Gene Ontology (GO) and Kyoto Encyclopedia of Genes and Genomes Pathway Enrichment (KEGG) Analysis

Based on BMLs- linked DEGs, the Gene Ontology (GO) enrichment (Supplementary Table S3), and Kyoto Encyclopedia of Genes and Genomes Pathway Enrichment (KEGG) (Supplementary Table S4) were evaluated and visualized using the GOplot (ver.1.0.2) and ggplot2 (ver.3.3.2) in R, respectively. The biological process (BP), cellular component (CC), and molecular function (MF) terms were annotated, respectively. Adjusted *P*-value < 0.05 was set as the cutoff criterion.

### Upset plots

To further screen hub genes of BMLs from BMLs-specific DEGs, we verified the DEGs through different sources (Supplementary Table S5-S6), such as independent transcriptomic datasets (EGAS00001004476, GSE51588) [11, 12], and OA risk genes identified by large-scale Genome Wide Association Study (GWAS) [23–25]. An UpSet plot was used to present the results. (https://github.com/hms-dbmi/UpSetR/).

### Construction of Protein–Protein Interaction (PPI) Network

The candidate hub genes were submitted into the Search Tool for the Retrieval of Interacting Genes (STRING 11.5, https://string-db.org/), including a series of PPI relationships. With the confidence score > 0.4 as the filter criteria, the PPI network was constructed and visualized using Cytoscape (ver3.9.0).

### Receiver operating characteristic (ROC) analysis

Through an independent subchondral bone RNA microarray profile, we performed ROC analysis and estimated the area under the curve (AUC) to confirm the diagnostic efficacy of possible hub genes for BMLs (E-MTAB-5564). AUC >0.7 was regarded high predictive efficacy (*P* value<0.05, R package “pROC” ver.1.18.0. ROC curves were compared against each other using “DeLong” test [26].

### Construction of TF & competing endogenous (ceRNA) network

To explore the transcriptional regulation involved in BMLs, TF-mRNA (hub genes) interactions were predicted combing with four open-accessed TF databases (Supplementary Table S7-S8) such as ENCODE (Encyclopedia of DNA Elements, https://www.encodeproject.org), ChEA3 (ChIP-X Enrichment Analysis 3, https://amp.pharm.mssm.edu/ChEA3), JASPAR 2020 (http://jaspar.genereg.net), and TRRUST v2 (Transcriptional Regulatory Relationships Unraveled by Sentence-based Text mining, www.grnpedia.org/trrust).

Similarly, ceRNA (mRNA-miRNA-lncRNA) networks were constructed. The miRNAs were predicted by open-accessed databases such as miRDB (http://www.mirdb.org/), tarBase v8 (https://carolina.imis.athenannovation.gr/diana_tools/web/index.php?r=tarbasev8%2Findex), miRTarbase 2020 (https://mirtarbase.cuhk.edu.cn), starBase v3.0 (http://starbase.sysu.edu.cn), and TargetScan human 7.0 (http://www.targetscan.org). The results were further verified by independent miRNA profiles [27, 28] related to subchondral bone (Supplementary Table S9-10). Consequently, the miRNA-lncRNA interactions were predicted by public databases such as lncBase v3(https://diana.e-ce.uth.gr/lncbasev3) and starBase v3.0 (https://starbase.sysu.edu.cn/index.php), and then validated by lncRNA profiles (EGAS00001004476) of subchondral bone in OA (Supplementary Table S11). The Sankey diagrams were used to present the results.

### Identification of cell type composition in subchondral bone by deconvolution analysis from transcriptome data

#### Acquisition of Publicly available single cell RNA-sequencing data and signature matrix of “subchondral bone”

Open-accessed scRNA-seq data from 4 cases of human femoral head with hip OA were collected (GSE169396) and one sample without clear information was excluded. The signature matrix was generated, and cell-types were obtained by integrating typical markers and differential expression using SingleR(ver.1.2.4) [29].

#### Generation and validation of a “subchondral bone” deconvolution signature matrices in MuSiC and MCP-counter

The cell type composition of the present bulk-seq of subchondral bone was first characterized by MuSiC deconvolution method [16], which was exploited by Wang *et.al* and enabled transferring cell type-specific gene expression information from scRNA-seq to bulk RNA-seq (https://xuranw.github.io/MuSiC/articles/MuSiC.html). Kruskal-Wallis test was performed to evaluate the differences between groups in each cell-type proportion. Co-expression analysis between hub genes and proportions of each cell-type was performed using Spearman’s correlation analysis by function “*cor*” of R software. *P* value< 0.05 was considered to be statistically significant. Plots were generated using the R package “*corrplot*” (Supplementary Table S12).

We additionally performed MCP-counter algorithm (ver.1.2.0 in R), which could robustly quantify the abundance of cell populations based on transcriptomic dataset to validate cell type composition in both present bulk-seq and independent datasets (E-MTAB-5564) [13]. Gene signatures were selected as the customized ones [17] and exhibited in Supplementary Table S13. Due to different processing approaches, these two datasets were processed different orders of magnitude. To count “MCP-counter score” in normalized form, we performed “min-max normalization” [30], a kind of linear transformative algorithm, to transform the maximums into one (100%), the minimums into zero (0%) and the remaining scores were scaled accordingly.

### Statistical analyses

Statistical analyses were conducted using R statistical package (ver.4.0.0). The Kruskal-Wallis test were used for statistical analysis, with *p*<0.05 considered as significant.

## Results

### BML-specific Differentially Expressed Genes

Present Bulk-seq had generated ~195 million pairs of reads of predominantly mRNA (messenger RNA). Approximately ~35,000 genes were detected. Disparate expression profiles were visualized using heatmap representation and unbiased hierarchical clustering was applied to assess the likely similarity of data within each group. The OA samples and non-OA samples were clustered together, respectively (Fig. 2B). In addition, the OA-BML samples were distinguished from normal samples (Fig. 2C) or OA-NBML samples (Fig. 2D). Next, DEG analysis identified 86 significant BMLs-specific DEGs (79 up-regulated, 7 down-regulated, Fig. 2E-H, Supplementary Table S2).

### Functional Enrichment Analysis of BMLs-specific DEGs

To investigate the probable mechanism of BMLs-linked DEGs, Gene Ontology (GO) and Kyoto Encyclopedia of Genes and Genomes (KEGG) enrichment analyses were conducted. In GO analysis, the most important terms in biological process (BP), cellular component (CC) and molecular function (MF) were the extracellular matrix organization, collagen−containing extracellular matrix and extracellular matrix structural constituent, respectively (Supplementary Fig. 1A-B; Supplementary Table S3), indicating the possible associations with osteosclerotic lesions. On the other hand, one of the most significant KEGG pathway (Supplementary Table S4) was the Wnt signaling pathway (Supplementary Fig. 1C), which is widely known as the consequence of extraordinary mechanical stress.

### Potential Small Molecular Drugs for BMLs

To screen candidate compounds targeting BMLs through BMLs-specific DEGs, a total of 41 associated compounds and the top 10 compounds were identified by CMap analysis, (Supplementary Fig. 1D). Additionally, the 3D chemical structures of the top 3 compounds were selected according to the scoring and exhibited in Supplementary Fig. 1E.

### Identification for BMLs-specific Hub Genes

**Fig. 3:**
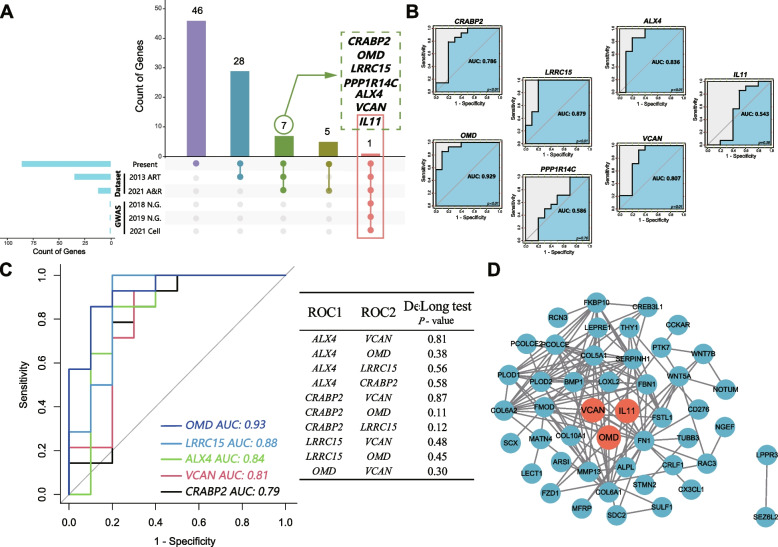
Identification for hub genes of BMLs. **A** Overlapping number of differential expression genes (DEGs) across the different sources. The Upset plot demonstrates the shared DEGs across different sources. Colored bars in the y-axis represented the total number of overlapping DEGs under different conditions. Blue bars in the x-axis represented the total number of DEGs included in each source, connected by the red dots in the body of the plot. **B** ROC curve analysis was performed for each potential hub gene. AUC scores and *P*-value were exhibited, respectively. **C** Left panel: ROC curves compare the AUCs for genes of good prediction value (AUC>0.7,* P* value<0.05 with DeLong method; Right panel: Comparison for each pair of genes.* P* value>0.05 was considered significant. **D** PPI network of the DEGs was constructed and core genes were highlighted in red

Seven potential hub genes of BMLs, including *CRABP2, OMD, LRRC15, PPP1R14C*, *ALX4*, *VCAN*, and *IL11* were identified by overlapping counts of DEGs across present bulk-seq and two open-accessed transcriptomic datasets from subchondral bone (Fig. 3A, Supplementary Table S5-S6). Of note, *IL11* was the merely overlapped gene among OA risk genes that were identified by GWAS.

To further evaluate the diagnostic efficiency of hub genes for subchondral lesions, the AUCs of potential hub genes were examined by ROC analysis (Fig. 3B) in an independent transcriptomic dataset (E-MTAB-5564). Among them, *OMD* (AUC=0.929), *LRRC15* (AUC=0.879), *ALX4* (AUC=0.836), *VCAN* (AUC=0.807) and *CRABP2* (AUC=0.786) exhibited favorable efficiency. *PPP1R14C* (AUC=0.586) and *IL11* (AUC=0.843) exhibited a moderate efficiency. Furthermore, the AUCs were compared with each other by “DeLong” method, and they exhibited similar diagnostic efficiency with each other (Fig. 3C).

The PPI network analysis was implemented based on these 7 DEGs. A total of 47 nodes and 140 edges were identified, and the unconnected nodes in the PPI network were removed. After that, *VCAN*, *IL11* and *OMD* were remained (Fig. 3D, in red).

Regarding *IL11,* it consistently demonstrated differential expression in the present bulk-seq analysis, previous transcriptomic datasets of subchondral bone and OA GWAS data. It was also identified by PPI analysis. For *VCAN*, it was not only screened out by overlapped DEGs in the present bulk-seq and transcriptomic datasets, but also identified by PPI. Finally, *VCAN* was validated by ROC curve analysis in an independent transcriptomic dataset [13]. In contrast, the osteomodulin (*OMD*) was excluded because it was unexpectedly found to be significantly reduced in subchondral bone of OA patients [31].

Furthermore, we applied immunofluorescence to detect the expression of IL-11 in BML, NBML and normal samples of subchondral bone. The upregulation of IL-11 was observed in BML sample, meanwhile, IL-11 was absent in normal and NBML samples.

Taken together, *IL11* and *VCAN* were identified as the core hub genes of BMLs.

### Cell Type Composition in Subchondral Bone

**Fig. 4:**
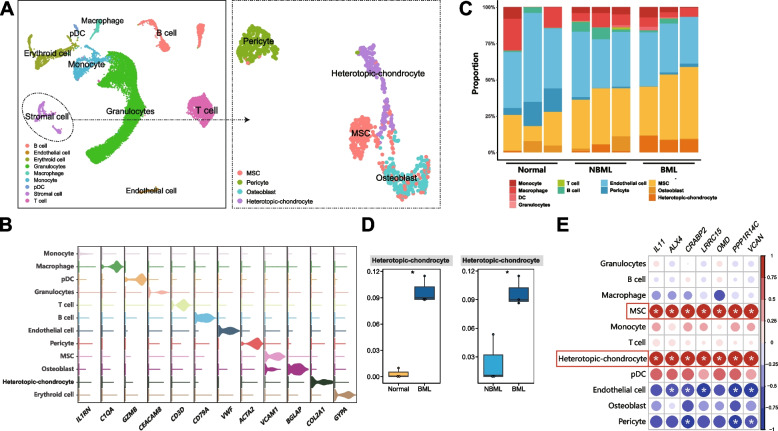
MuSiC for cell-type composition analysis of bulk RNA-sequencing. **A** UMAP of single-cell RNA sequencing, with each cell color-coded for the associated cell type. 12 kinds of cell types were signatured, including 9 kinds of cell types in preliminary, while stromal cell was further divided into 4 subpopulations. **B** Violin plots of the expression of markers for each cell type. **C** A total of 11 kinds of cell types were recognized in present bulk-seq. Exhibition of cell proportions with the estimated proportions by MuSiC for all subchondral bone samples. **D** For heterotopic-chondrocyte, the cell proportion demonstrated more highly accounted in OA-BML group than that in non-OA group or OA-NBML group, comparing by Kruskal-Wallis test . *P* value < 0.05 was considered as significant difference. **E** Correlation analysis between the potential hub-genes of BMLs and distinct cell types, by Spearman’s correlation analysis. The correlation coefficients were shown in each cell type indicated by shade of color in dots, while red color represented positive correlation and blue color was the opposite. Correlation coefficient |R| > 0.5, *p* < 0.05 was considered statistically significant. The asterisk (*) represents *p*<0.05

To explore the microenvironment of subchondral bone, the MuSiC was firstly performed to deconvolve present bulk-seq at single-cell level. According to a total of 12 kinds of cell types in reported scRNA-seq (GSE169396), we identified 11 kinds of cell types in present bulk-seq, except for erythroid cell (Fig. 4A-B). The corresponding proportions of each cell type of individual samples were exhibited in Fig. 4C and Supplementary Table S12. Comparisons of cell type between groups were performed, and substantial differences of the heterotopic-chondrocytes were determined not only between OA-BML and OA-NBML, but also between OA-BML and non-OA, respectively (Fig. 4D), indicating aggravated heterotopic chondrogenesis in BMLs.

To evaluate the correlation between potential hub genes and each cell type, the correlation analysis was applied in OA-BML, revealing that the prospective hub genes of OA-BML displayed a consistent correlation to heterotopic-chondrocyte and mesenchymal stem cell (MSC), indicating their crucial roles in subchondral bone remodeling. Moreover, *IL11* and *VCAN* exhibited tight connection to heterotopic-chondrocyte and MSC (Fig. 4E).

**Fig. 5:**
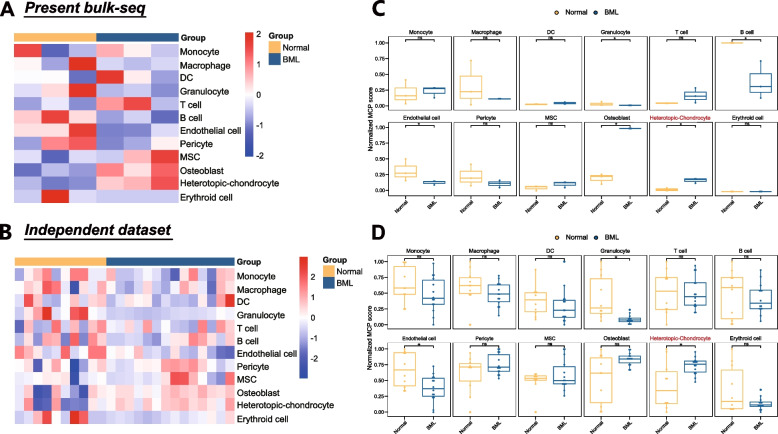
MCP-counter algorithm revealed the cell type composition of transcriptome. **A**-**B** MCP-counter algorithm: the heatmaps showed the absolute abundances of cell subsets from (**A**) present bulk-seq and (**B**) independent transcriptome dataset. **C**-**D** The boxplots compared the MCP-counter scores for different cell types in normal and lesioned subchondral bone tissues from (**C**) present bulk-seq and (**D**) independent transcriptome datasets by Kruskal-Wallis test, respectively. *P* value < 0.05(*) was considered as significant difference, while “ns” represented not significant

Additionally, we tried to verify the robustness of cell type composition by another deconvolution algorithm, which named “MCP-counter”, in both present bulk-seq (Fig. 5A, Supplementary Fig. 2) and an independent transcriptomic dataset (Fig. 5B). MCP-counter analysis identified similar cell-type composition to MuSiC and demonstrated that the clusters of heterotopic-chondrocyte and osteoblast were consistently more abundant in OA-BML than those in controls (Fig. 5C-D, Supplementary Table S14, S15). These findings suggested that the cell type of heterotopic-chondrocyte might be one of the characteristic features in OA-BML.

### Exploration of Regulatory Network of *IL11* and *VCAN*

We then tried to explore the transcriptional regulatory mechanisms of *IL11* and *VCAN.* The transcription factors (TFs)-target interaction was identified by constructing TF-mRNA network (Supplementary Table S7, S8). The possible *VCAN-*targeting TFs were predicted using four open-accessed TF databases (ENCODE, ChEA, JASPAR, and TRRUST), and the *TP53* was the only TF overlapped in at least two databases. Similarly, the *EGR1* was identified as the *IL11-*targeting TF.

Next, the *VCAN-*targeting and *IL11-*targeting miRNAs were predicted by using open-accessed databases (miRDB, tarBase, miRTarbase, starBase, and TargetScan), respectively. For *IL11*, a total of 574 miRNAs were predicted, and then verified by two publicly available miRNA differential expression profiles related to subchondral bones (Supplementary Table S9). We identified that has-miR-424-5p and has-miR-470-5p were overlapped in at least one database, coupling with more than one miRNA profile. Similarly, a total of 1053 *VCAN*-targeting miRNAs were predicted, and both has-miR-424-5p and has-miR-4261 were verified (Supplementary Table S10).

**Fig. 6:**
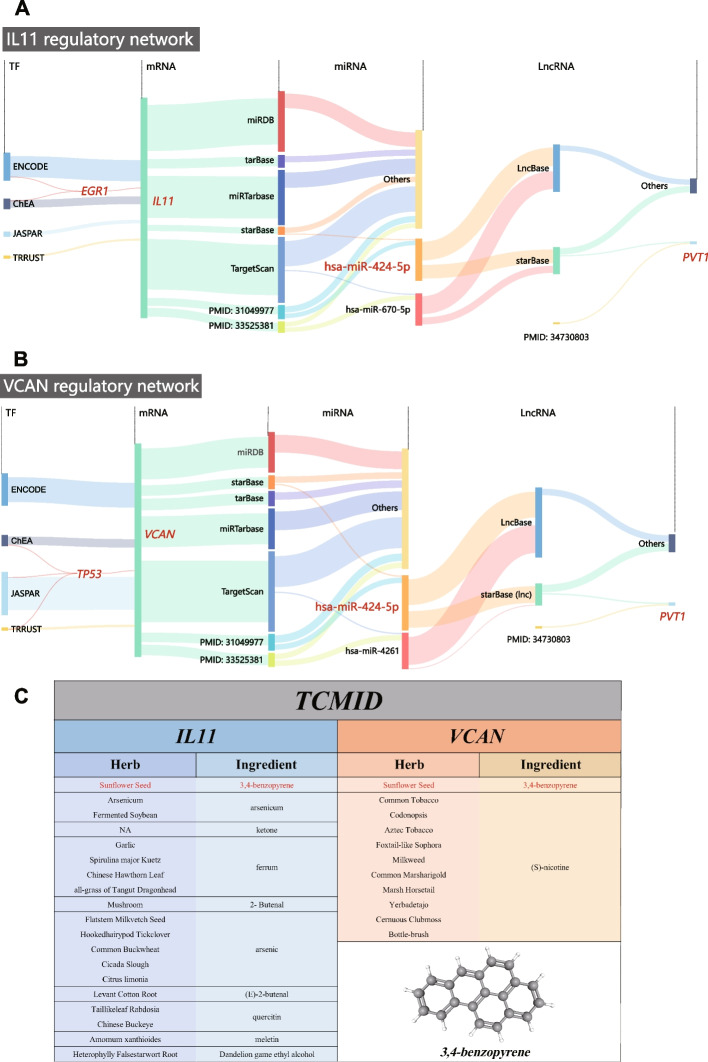
Exploration of Regulatory Network and Screening Potential Herbal ingredients targeting hub genes of BMLs. **A**-**B** Sankey diagrams were generated to visualize the “TF-mRNA” network combining with ceRNA network (mRNA/miRNA/lncRNA), which were established in *IL11* (**A**) and *VCAN* (**B**), as the hub genes of BML, respectively. The crucial molecules constituting network were highlighted in red. **C** Using TCMID (Traditional Chinese Medicine Integrated Database), potential ingredients and related herbs that target hub genes (*IL11, VCAN*) of BML were predicted and demonstrated. The bottom right demonstrated 3D chemical structure of the ingredient as “*3,4-benzopyrene*”

Subsequently, miRNA-targeting lncRNAs were predicted by using open-accessed databases lncBase and starBase. A total of 273 and 1012 lncRNAs were predicted for has-miR-424-5p and has-miR-4261, respectively. After that, they were validated by the reported lncRNAs profile of subchondral bone(EGAS00001004476). We then identified lncRNA *PVT1* as the only lncRNA that overlapped in the predictive database and lncRNA profile for miR-424-5p, whereas none for has-miR-4261. The Sankey diagrams showed the complete regulatory network, combing with “TF-mRNA” and “mRNA-miRNA-lncRNA” ceRNA network (Fig. 6A-B).

Finally, we explored if Chinese traditional herbs and ingredient could target *IL11* or *VCAN* by database of TCMID. Notably, ingredient “*3,4-benzopyrene*”, extracted from the herb named “*Sunflower Seed*”, was identified targeting both hub genes (Fig. 6C).

## Discussion

In the present study, we performed hierarchical clustering to determine the distinct expression pattern between OA-BML, OA-NBML and non-OA samples. Next, we screened for BML-specific DEGs to reveal gene expression pattern of BMLs. What’s more, *IL11* and *VCAN* were identified as core hub genes of BMLs*,* verified through DEGs from present bulk-seq and reported transcriptomic datasets. Subsequently, ROC curve analyses in independent datasets of subchondral bone [13] and the construction of PPI networks were performed to further verified the core hub genes. To uncover cell type compositions in subchondral bone, two novel bioinformatic algorithms were utilized to deconvolute present bulk-seq data based on open-accessed scRNA-seq data from hip OA. We obtained a total of 11 cell types from present bulk-seq and verified them in independent datasets. We further determined that heterotopic-chondrocytes processed significantly difference in the comparisons of OA-BML versus OA-NBML, and OA-BML versus non-OA samples, respectively. Finally, regulatory mechanisms of *IL11* and *VCAN* including TF-mRNA network and ceRNA network were predicted and validated. As consequences, the networks of “*EGR1*/*IL11*/has-miR-424-5p/lncRNA PVT1” and *TP53*/*VCAN*/has-miR-424-5p/lncRNA PVT1” were determined.

Accumulating studies have emphasized the crucial roles of BMLs on the progression of OA [13, 32]. However, the epigenetic regulatory mechanism and cell-type compositions of subchondral BMLs in OA are poorly understood. To the best of our knowledge, this is the first study to explore the hub genes, regulatory network of subchondral BMLs in OA. More importantly, we confirmed that *IL11* and *VCAN* were core hub genes in BMLs. The “*has-miR-424-5p/lncRNA PVT1”* was determined as crucial network, targeting *IL11* and *VCAN*, respectively. We also profiled the microenvironment of BMLs using deconvolution approaches at the single-cell level, while the cluster of heterotopic-chondrocyte was discovered abundant in BMLs and positively correlated with the expression of hub genes.

As far as we know, *VCAN*, also termed as versican, is the first time to be identified as a hub gene of BMLs. Versican is an extracellular matrix (ECM) component [33] secreted by stromal cells, which involves in cell adhesion, proliferation, migration, and extracellular matrix assembly [34]. The aberration of versican plays critical roles in fibroproliferative diseases, such as exacerbating degradation of myocardial cells (Heart failure) [33], increasing activation of hepatic stellate cells (Hepatic fibrosis) [35] and thickening tunica intima (Ischemic cerebral small-vessel disease) [36]. Moreover, versican has been discovered upregulated in cartilage [37] and medial meniscus of OA patients [38], indicating its intrinsic connection to OA. Yan *et.al* reported that *VCAN* was a direct target of miRNA and induced BMSC (bone-derived MSC) senescence [39]. In present study, we identified *VCAN* as a core hub gene of BMLs, and it was significantly associated with MSC and heterotopic-chondrocyte at single-cell level, suggesting that *VCAN* might be involved in aberrant ECM and osteosclerosis of BMLs. *IL11* was also identified as a core hub gene of BMLs by comprehensive evaluations in present stud*y.* It was the unique one of seven potential hub genes of BMLs that was identified as OA risk gene from GWAS (Fig. 3A). Previous GWAS analyses exhibited the correlation between *IL11* and hip OA, rather than knee OA [23, 24]. *IL11* was detected upregulating in both OA subchondral bone (knee and hip) and cartilage [10], as well as in synovial fluid [40]. Interestingly, *IL11* was reported to facilitate fibrosis and inflammation in cardiovascular fibrosis [41] and rheumatoid arthritis [42]. In addition, *IL11* could also act on bone marrow-derived stem cells [43]. Moreover, IL-11 also play an important role in bone formation, remodeling and resorption [44]. It has been reported that IL-11 is an important regulator of osteoblast differentiation by regulating canonical Wnt/β-catenin signaling [45]. Studies had showed that IL-11 stimulates osteoclastogenesis by both RANKL and RANKL-independent mechanism [46, 47]. Additionally, IL-11 was reported to increase the degree of mineralized nodule formation in human periodontal ligament stem/progenitor cell line [48]. Our research determined that IL-11 was upregulated in BMLs, which might accelerate the progression of bone remodeling and mineralization alternations in OA. Our results (Fig. 4) further demonstrated that *IL11* closely linked to heterotopic-chondrocyte and MSC, indicating that *IL11* might be involved in inducing heterotopic-chondrocyte, which is the abnormal chondrogenic differentiation from MSC in BMLs.

Studies have found that traditional Chinese medicines (TCM) could treat OA [49, 50]. Therefore, we utilized the Traditional Chinese Medicine Integrated Database for TCM targeting *IL-11* and *VCAN*. In this study, we found that 3,4 benzopyrene, an ingredient from “sunflower seed”, can target both *IL11* and *VCAN*. The 3,4 benzopyrene, a polycyclic aromatic hydrocarbon (PAH), which is one of the main ingredients in cigarettes, was identified. It is an agonist of the aryl hydrocarbon receptor (AhR) [51]. Previous studies have revealed that it suppressed the BMP2-induced osteogenic differentiation of mesenchymal stem cells [52], which suggests that it might inhibit the abnormal bone remodeling of BMLs by targeting *IL-11* or *VCAN*. The findings provided novel therapeutic strategies for knee OA patients with BMLs. Further experiments of these TCMs are required afterwards to explore therapeutic effects both in vivo and vitro.

The role of non-coding RNA in BMLs remains largely uncovered. In current study, we identified hsa‐miR‐424‐5p and lncRNA *PVT1* as the critical ceRNA networks to target *IL-11* and *VCAN.* Xia *et.al* [53] found that hsa‐miR-424‐5p was downregulated in cartilage-derived MSC from degraded cartilage of OA, and Wang *et.al* [27] also reported that hsa‐miR‐424‐5p was downregulated in OA serums. Although our finding of the hsa‐miR‐424‐5p is consistent with above expression patterns, the specific function of hsa‐miR‐424‐5p in MSC of subchondral bone and whether hsa‐miR‐424‐5p is involved in chondrogenic differentiation are still unknown. Our findings indicate that downregulation of hsa‐miR‐424‐5p might reduce the inhibition of *IL-11* and *VCAN* in BMLs. LncRNA *PVT1* is located at 8q24.21 of chromosome and has been previously reported in metabolic disorder [54]. Moreover, upregulation of lncRNA *PVT1* has been observed in OA cartilage, and our findings might bring new insight of lncRNA *PVT1* into the regulation mechanisms of subchondral bone.

Recent studies have focused on individual cell type in subchondral bone, such as osteoblast precursor cell [55] and pre-osteoblast [56]. However, the microenvironment of subchondral bone remained unrevealed, and we tried to characterize the cell type compositions at the single-cell level by utilizing newly established deconvolution algorithms of MuSiC [15] and MCP-counter [16]. They were reported to estimate proportion of cell types in cancers [15] and rheumatic arthritis [17] from transcriptomic data. Interestingly, our study found that the heterotopic-chondrocyte was consistently and significantly abundant in OA-BML than that in OA-NBML or non-OA group, which was verified by two algorithms and independent datasets. Furthermore, the hub genes of BMLs were also significantly associated with the heterotopic-chondrocyte and MSC. The finding indicates that *IL11* and/or *VCAN* might be overexpressed by paracrine of heterotopic-chondrocyte or autocrine of MSC itself, promoting aberrated chondrogenic differentiation, instead of speculation of disordered chondrocyte migration [57]. On the other hand, erythroid cell was absent in present and validated datasets. Bulk-seq was more accessible and economical than scRNA-seq. In the present study, we exhibited the feasibility of combing deconvolution methodologies to efficiently characterize the cellular heterogeneity of subchondral bone using bulk-seq.

This study has some limitations. First, there was a limited sample in present study. To mitigate potential heterogeneity bias, we screened hub genes of BMLs by comparing with a series of RNA transcriptomic datasets of subchondral bone and the list of OA risk genes from GWAS, and subsequently verified them using independent RNA transcriptomic profiles. We believe that the results were fairly robust. Second, the scRNA-seq exclusive for subchondral bone is still absent. However, we performed newly established deconvolution algorithms based on signatures of scRNA-seq from OA femoral head, which was the closest approximate dataset at present, with similar composition of subchondral bone histologically, such as cancellous bone and bone marrow tissue. Third, the individual heterogeneity of bulk-seq and scRNA-seq may have impact on the correlation analysis. Last, in-depth biological verification has yet to be done and further verifications to reveal regulatory mechanisms in vitro and in vivo are needed.

## Conclusion

In conclusion, *IL11* and *VCAN* were identified as the core hub genes of BMLs, and their underlying molecular networks were determined as well. We profiled the characteristics of subchondral bone at single-cell level, and determined that the heterotopic-chondrocyte was abundant in BMLs and was closely linked to *IL11* and *VCAN*. Our study provides new insights into the cellular and molecular underpinning of BMLs, and may contribute to the development of novel therapeutic strategies.

### Supplementary Information

**Additional file 1:** **Supplementary Table S1.** Detailed information of nine samples from six donors in present study. **Supplementary Table S2.** Overlapping differentially expressed genes between two differentially expressed genes list  with similar direction of effect. **Supplementary Table S3.** GO analysis on differentially expressed genes (n=86 genes) in OA subchondral bone marrow lesion (top 10 dispalyed). **Supplementary Table S4.** KEGG analysis on differentially expressed genes (n=86 genes) in OA subchondral bone marrow lesion (top 7). **Supplementary Table S5.** Overlapping differentially expressed genes between four differentially expressed genes  with same expression trend. **Supplementary Table S6.** Summary of OA risk genes reported in genome-wide association studies. **Supplementary Table S7.** IL11-TF network prediction. **Supplementary Table S8.** VCAN-TF network prediction. **Supplementary Table S9.** Predicted miRNAs binding to IL11 through databases and publisheddatasets  (top 20 displayed). **Supplementary Table S10.** Predicted miRNAs binding to VCAN through databases and published datasets  (top 20 displayed). **Supplementary Table S11.** Top 20 predicated lncRNA binding to hsa-miR-424-5p , hsa-miR-670-5p and has-mir-4261 through databases. **Supplementary Table S12.** The estimated cell proportions of present bulk RNA-seq by MuSiC. **Supplementary Table S13.** Customized signature matrix of scRNA-seq for MCP counter. **Supplementary Table S14.** Cellular abundance of present bulk RNA-seq predicted by MCP counter. **Supplementary Table S15.**Cellular abundance of independent subchondral bone RNA microarray profile(E-MTAB-5564) predicted by MCP counter.

**Additional file 2:** **Supplementary Fig 1.** Function analysis of BML-specific DEGs. (A-B) Gene Ontology (GO) enrichment analysis. Y-axis, negative log-adjusted p-value; x-axis, z-score; Bubble area positively correlated with counts of gene numbers in indicated terms. Green, biological process (BP); pink, cellular component (CC); blue, molecular function (MF). Adjust P-value＜0.05 (orange cross-line) was considered significant. (C) Results of Kyoto Encyclopedia of Genes and Genomes (KEGG) Pathway Enrichment Analysis. (D) Results of CMap analysis, sorted by p-value from small to large order. (E) The 3D chemical structure of the top three small molecule drugs for BMLs (from left to right respectively: Ampicillin, Anisomycin, Astemizole).

**Additional file 3:** **Supplementary Fig 2. **HE and immunofluorescence staining of Normal (A), NBML (B), and BML (C) subchondral bone. Subchondral bone samples were stained for HE and immunofluorescence of IL-11 (red), with DAPI (blue) being used for nuclear counterstaining.

**Additional file 4:** **Supplementary Fig 3.** Cell type composition fraction. (A) Difference between OA-BML and non-OA group of each cell type. (B) Difference between OA-BML and OA-NBML group of each cell type. Kruskal-Wallis test was performed and *p < 0.05 was considered statistically significant.

## Data Availability

Datasets (EGAS00001004476, E-MTAB-5564, GSE51588, GSE169396) analyzed in present study were publicly available. The RNA sequencing data can be found online in NCBI https://www.ncbi.nlm.nih.gov/sra/PRJNA800573.

## Abbreviations

AUC: Area under the curve
BML: Bone marrow lesion
BP: Biological process
Bulk-seq: Bulk RNA-sequencing
ceRNA: Competing endogenous RNA
CC: Cellular component
ChEA: ChIP-X Enrichment Analysis
CMap: Connectivity Map
DEGs: Differentially expressed genes
ENCODE: Encyclopedia of DNA Elements
FC: Fold change
GWAS: Genome-wide association study
GO: Gene Ontology
KEGG: Kyoto Encyclopedia of Genes and Genomes
MCP-counter: Microenvironment cell population counter
MF: Molecular Function
MOAKS: MRI Osteoarthritis Knee Score
MRI: Magnetic Resonance Imaging
MSC: Mesenchymal stem cell
MuSiC: MUlti-Subject SIngle Cell deconvolution
NBML: non-BML
OA: Osteoarthritis
PPI: Protein–Protein Interaction
ROC: Receiver operating characteristic
scRNA-seq: Single-cell RNA sequencing
TF: Transcript factor
TCM: Traditional Chinese Medicine
TCMID: Traditional Chinese Medicine Integrated Database
TKR: Total knee replacement
TRRUST: Transcriptional Regulatory Relationships Unraveled by Sentence-based Text mining
