# Supplementary material for: Comprehensive analysis on subchondral bone marrow lesions of human osteoarthritis by integrating bulk and single-cell transcriptomes
Source: BMC Musculoskelet Disord. 2023 Aug 25;24:677. doi: 10.1186/s12891-023-06676-4 (PMC10463447; doi:10.1186/s12891-023-06676-4)
Supplement: Supplementary file 2 — Additional file 2: Supplementary Fig 1. Function analysis of BML-specific DEGs. (A-B) Gene Ontology (GO) enrichment analysis. Y-axis, negative log-adjusted p-value; x-axis, z-score; Bubble area positively correlated with counts of gene numbers in indicated terms. Green, biological process (BP); pink, cellular component (CC); blue, molecular function (MF). Adjust P-value＜0.05 (orange cross-line) was considered significant. (C) Results of Kyoto Encyclopedia of Genes and Genomes (KEGG) Pathway Enrichment Analysis. (D) Results of CMap analysis, sorted by p-value from small to large order. (E) The 3D chemical structure of the top three small molecule drugs for BMLs (from left to right respectively: Ampicillin, Anisomycin, Astemizole). [file 12891_2023_6676_MOESM2_ESM.pdf]

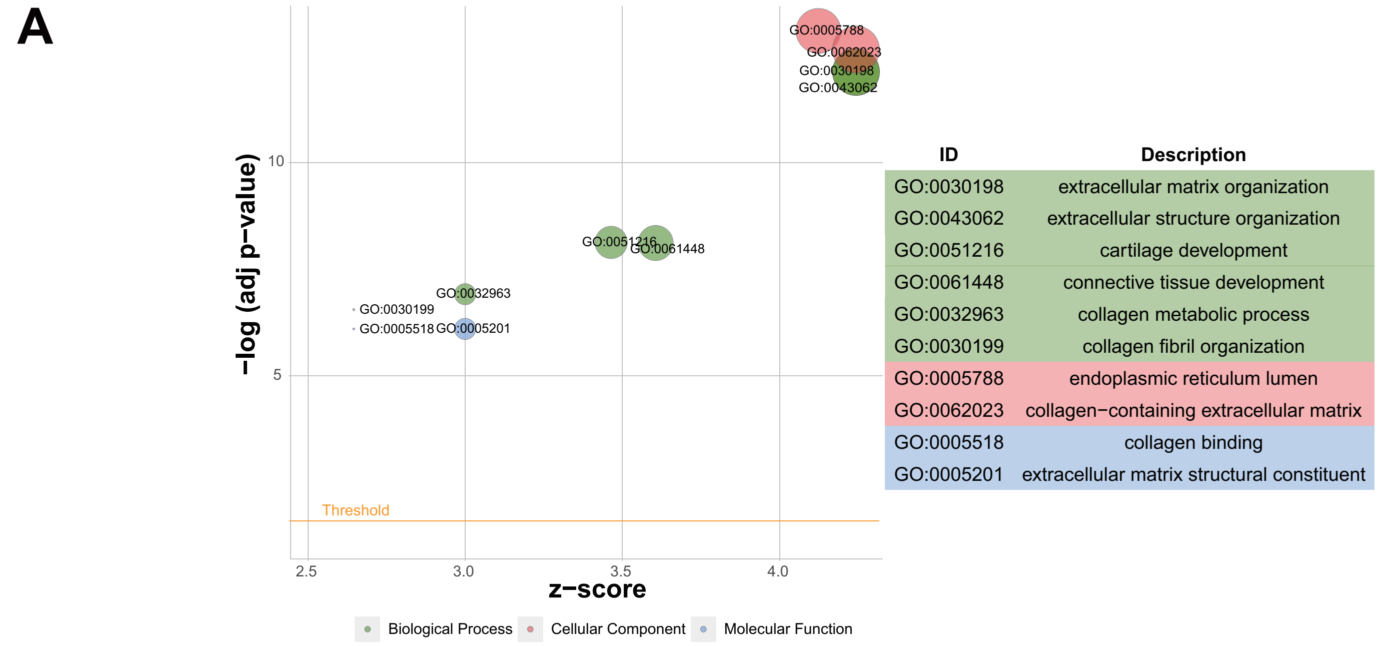

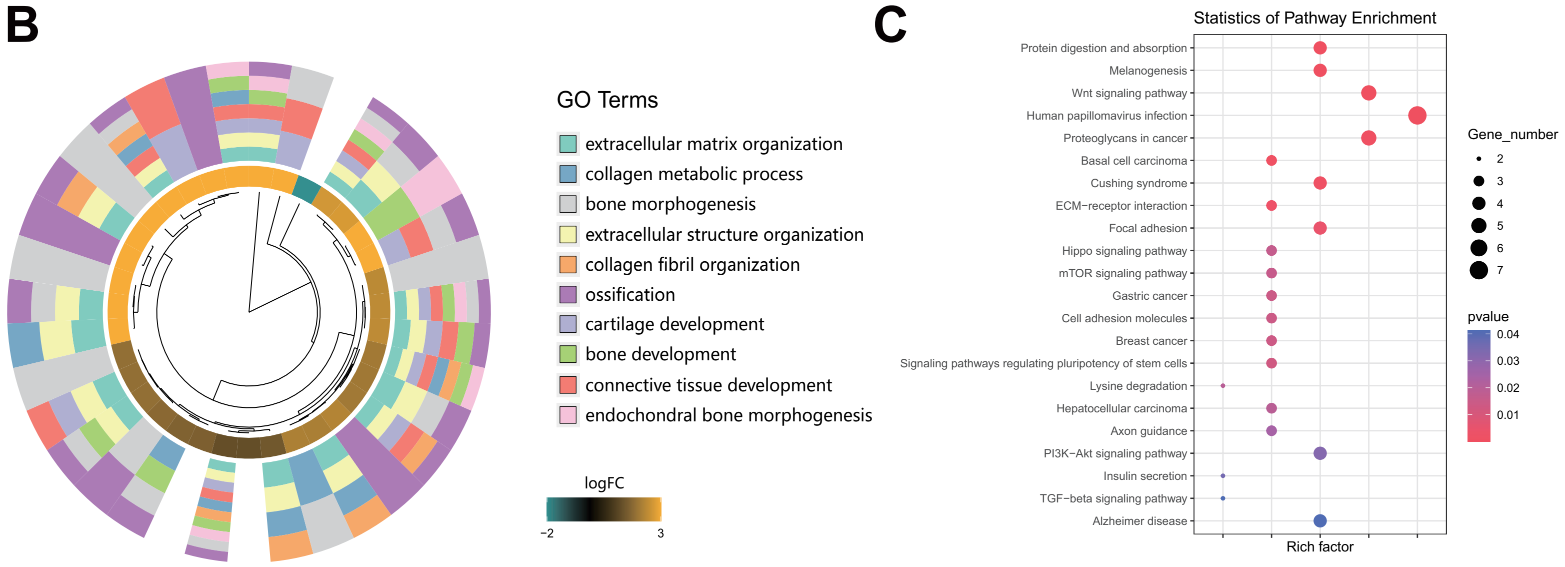

D

CMap name	Mean	*n*	Enrichment	*p*
Ampicillin	-0.469	4	-0.81	0.00257
Anisomycin	-0.286	4	-0.794	0.00356
Astemizole	-0.421	5	-0.706	0.00477
Indoprofen	-0.412	4	-0.78	0.00483
Pizotifen	-0.41	4	-0.759	0.00692
Prestwick-642	-0.24	4	-0.74	0.00891
Ascorbic acid	-0.394	4	-0.729	0.01082
Cicloheximide	-0.298	4	-0.715	0.01337
Tamoxifen	-0.296	7	-0.548	0.01649
Thiamazole	-0.166	6	-0.587	0.01738

E

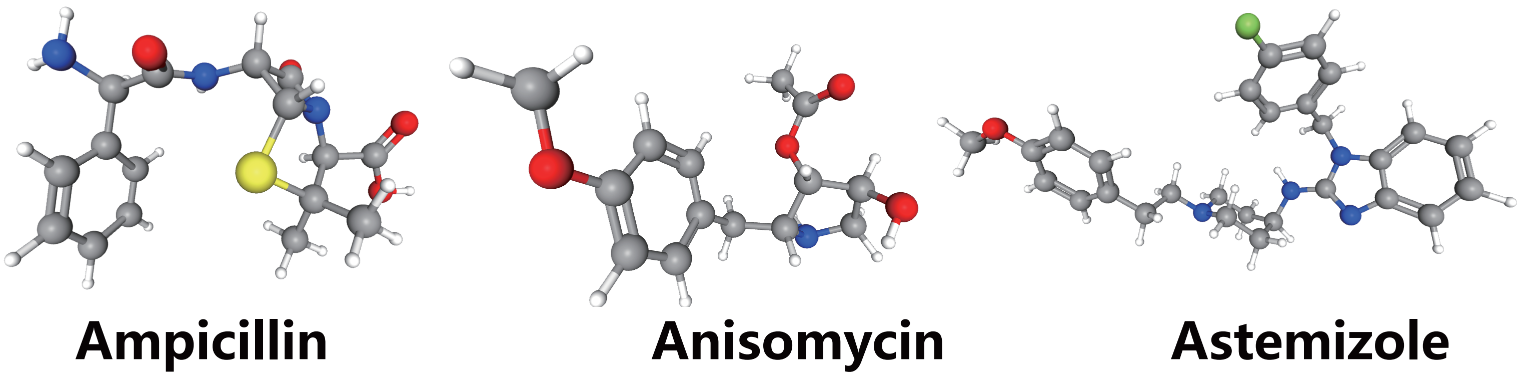

Supplementary Fig. 1 Function analysis of BML-specific DEGs
(A-B) Gene Ontology (GO) enrichment analysis. Y-axis, negative log-adjusted p-value; x-axis, z-score; Bubble area positively correlated with counts of gene numbers in indicated terms. Green, biological process (BP); pink, cellular component (CC); blue, molecular function (MF). Adjust P-value＜0.05 (orange cross-line) was considered significant.
(C) Results of Kyoto Encyclopedia of Genes and Genomes (KEGG) Pathway Enrichment Analysis.
(D) Results of CMap analysis, sorted by p-value from small to large order.
(E) The 3D chemical structure of the top three small molecule drugs for BMLs (from left to right respectively: Ampicillin, Anisomycin, Astemizole).
