# Supplementary material for: Comprehensive analysis on subchondral bone marrow lesions of human osteoarthritis by integrating bulk and single-cell transcriptomes
Source: BMC Musculoskelet Disord. 2023 Aug 25;24:677. doi: 10.1186/s12891-023-06676-4 (PMC10463447; doi:10.1186/s12891-023-06676-4)
Supplement: Supplementary file 3 — Additional file 3: Supplementary Fig 2. HE and immunofluorescence staining of Normal (A), NBML (B), and BML (C) subchondral bone. Subchondral bone samples were stained for HE and immunofluorescence of IL-11 (red), with DAPI (blue) being used for nuclear counterstaining. [file 12891_2023_6676_MOESM3_ESM.pdf]

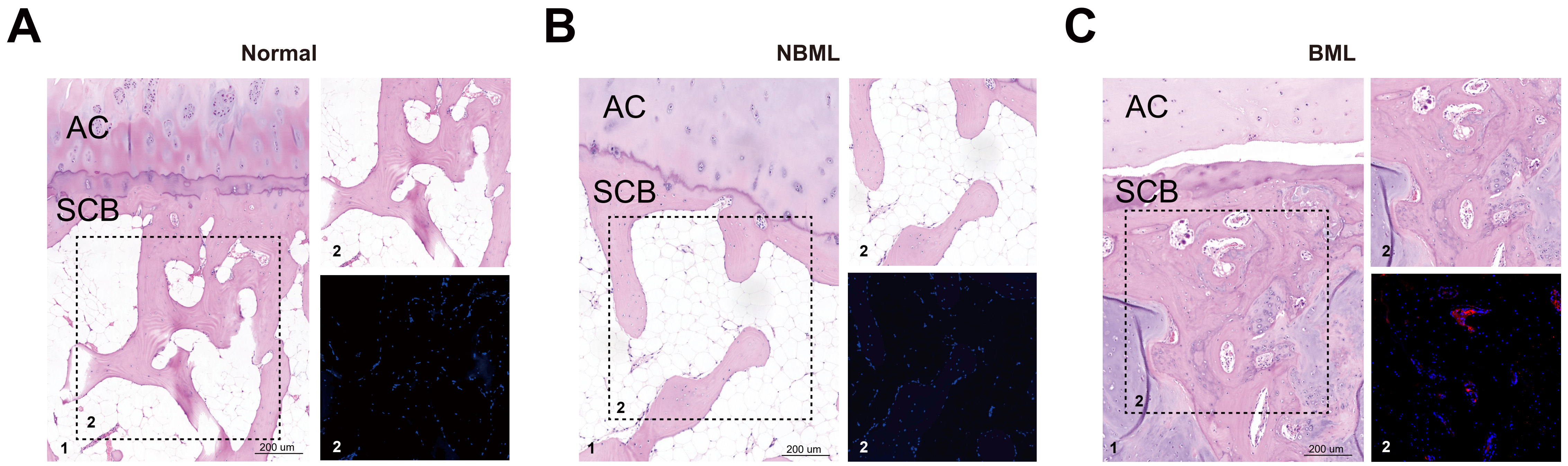

Supplementary Fig. 2 HE and immunofluorescence staining of Normal (A), NBML (B), and BML (C) subchondral bone. Subchondral bone samples were stained for HE and immunofluorescence of IL-11 (red), with DAPI (blue) being used for nuclear counterstaining.
