# Supplementary material for: Comprehensive analysis on subchondral bone marrow lesions of human osteoarthritis by integrating bulk and single-cell transcriptomes
Source: BMC Musculoskelet Disord. 2023 Aug 25;24:677. doi: 10.1186/s12891-023-06676-4 (PMC10463447; doi:10.1186/s12891-023-06676-4)
Supplement: Supplementary file 4 — Additional file 4: Supplementary Fig 3. Cell type composition fraction. (A) Difference between OA-BML and non-OA group of each cell type. (B) Difference between OA-BML and OA-NBML group of each cell type. Kruskal-Wallis test was performed and *p < 0.05 was considered statistically significant. [file 12891_2023_6676_MOESM4_ESM.pdf]

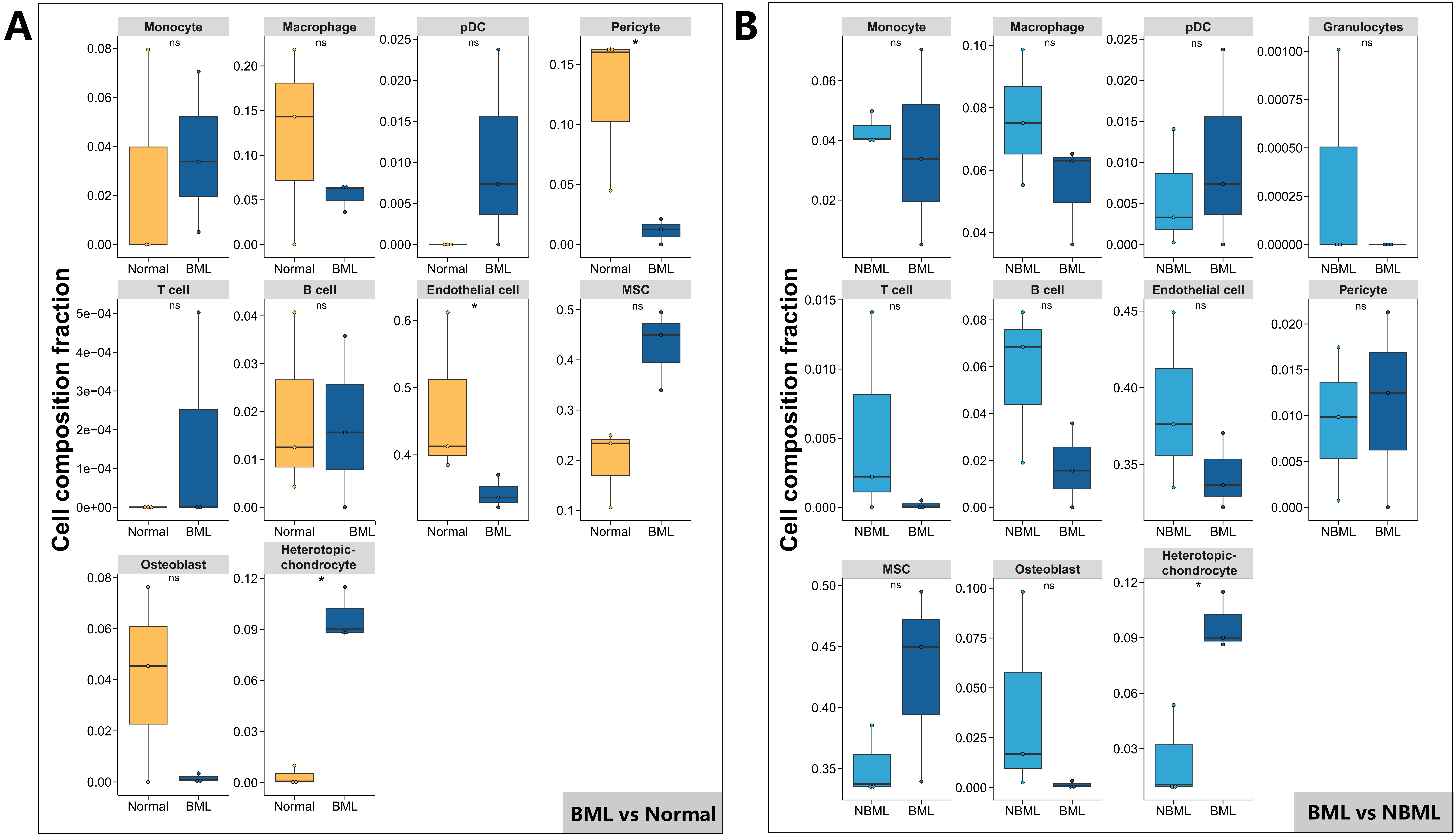

Supplementary Fig. 3 Cell type composition fraction

(A) difference between OA-BML and non-OA group of each cell type. (B) difference between OA-BML and OA-NBML group of each cell type. Kruskal-Wallis test was performed and $*p < 0.05$ was considered statistically significant.
